# Genetic deletion of Kvβ2 (AKR6) causes loss of muscle function and increased inflammation in mice

**DOI:** 10.3389/fragi.2023.1175510

**Published:** 2023-06-12

**Authors:** Ravikumar Manickam, Jazmine Virzi, Anish Potti, Feng Cheng, David W. Russ, Srinivas M. Tipparaju

**Affiliations:** ^1^ Department of Pharmaceutical Sciences, Taneja College of Pharmacy, University of South Florida, Tampa, FL, United States; ^2^ School of Physical Therapy and Rehabilitation Sciences, Morsani College of Medicine, University of South Florida, Tampa, FL, United States

**Keywords:** Kvβ2, KCNAB2, AKR6, sarcopenia, inflammation, skeletal muscle

## Abstract

The voltage-gated potassium channels (Kv) are complex ion channels with distinct roles in neurotransmission, electrical conductivity of the heart, and smooth and striated muscle functions. Previously, we demonstrated that deletion of Kvβ2 in mice results in decreased Pax7 protein levels, hindlimb muscles and body weights, and fiber type switching. In the present study, we tested the hypothesis that Kvβ2 regulates skeletal muscle function in mice. The young and old Kvβ2 knockout (KO) and wildtype (WT) mice were utilized to test the aging phenotype and skeletal muscle function. Consistent with our previous finding, we found a significant reduction in hindlimb skeletal muscles mass and body weight in young Kvβ2 KO mice, which was also significantly reduced in old Kvβ2 KO mice compared with age-matched WT mice. Forelimb grip strength, and the hindleg extensor digitorum longus (EDL) muscles force-frequency relations were significantly decreased in young and old Kvβ2 KO mice compared to age-matched WT mice. Analysis of transmission electron microscopy images of EDL muscles in young mice revealed a significant reduction in the sarcomere length for Kvβ2 KO vs. WT. Hematoxylin and eosin-stained tibialis anterior muscles cryosections displayed a significant decrease in the number of medium (2,000–4,000 µm^2^) and largest (>4,000 µm^2^) myofibers area in young Kvβ2 KO vs. WT mice. We also found a significant increase in fibrotic tissue area in young Kvβ2 KO mice compared with age-matched WT mice. Analysis of RNA Seq data of the gastrocnemius muscles (GAS) identified significant increase in genes involved in skeletal muscle development, proliferation and cell fate determination, atrophy, energy metabolism, muscle plasticity, inflammation, and a decrease in circadian core clock genes in young Kvβ2 KO vs. WT mice. Several genes were significantly upregulated (384 genes) and downregulated (40 genes) in young Kvβ2 KO mice compared to age-matched WT mice. Further, RT-qPCR analysis of the GAS muscles displayed a significant increase in pro-inflammatory marker Il6 expression in young Kvβ2 KO mice compared to age-matched WT mice. Overall, the present study shows that deletion of Kvβ2 leads to decreased muscles strength and increased inflammation.

## Introduction

The voltage-gated potassium (Kv) channels are transmembrane channels selective to K^+^ and belong to the larger ion channel families (Gutman et al., 2003). The Kv channels modulate action potential shape and duration in a variety of tissues, including skeletal and cardiac muscles (Nerbonne, 2016; Kilfoil et al., 2019). The human genome encodes 40 Kv channels that are classified into 12 groups based on their amino acid sequence homology (Kv1-12) with diverse physiological functions (Wulff et al., 2009). The α-subunits form homo- or heterodimers and can also associate with cytoplasmic β-subunits (Long et al., 2005). The voltage gated Kv channels (Kv1,4,7) influence the threshold and number of action potentials generated during depolarization or excitatory synaptic potentials. Whereas Kv2 and Kv3 modulate the duration and firing pattern of the action potentials. The kinetics of Kv channels also influence the action potentials generated. The Kv channels also play a role in calcium signaling, cell proliferation, migration, differentiation, cell death and maintenance of cell volume (Bachmann et al., 2020). Deficiency of Kv channels leads to neurological and metabolic disorders, cardiovascular and autoimmune diseases, cancer, and deafness (Glasscock et al., 2010; Leitner et al., 2011; Anderson et al., 2019; Trosclair et al., 2021). Therapeutically, the Kv channel activators or blockers alter the action potential’s firing pattern (Vyas et al., 2019).

Skeletal muscle is a major tissue comprising around 40% of the total body mass in humans. Skeletal muscle is also an active metabolic organ and helps in movement through contraction and relaxation of the myofibrillar proteins upon nervous stimulation. It has high tissue plasticity to adapt to varying conditions such as exercise, pathophysiological states, and aging (Manickam and Wahli, 2017). Sarcopenia, the age-associated decline in muscle mass and mechanical function, occurs in the context of chronic inflammation, mitochondrial dysfunction, metabolic deregulation, reduced stem cell number and function, and altered gene expression (Larsson et al., 2019). In a previous study, we demonstrated that loss of Kvβ2 leads to decrease muscle size and mass, and therefore in the present study we evaluated the effect of Kvβ2 deletion on muscle strength and impact in both young and aging mice.

The Kvβ subunits 1–3/KCNAB family of Kv channels belong to NADPH-dependent aldo-keto reductases (AKR) and bind to NADPH-cofactors. These Kvβ subunits form complexes with Kv1 and Kv4 channels α-subunits and modulates the gating and kinetics of Kv channels, membrane localization, and their redox sensitivity (Bähring et al., 2001; McCormack et al., 2002; Tipparaju et al., 2008). The expression of different Kvβ subunits such as Kvβ1.1–1.3, and Kvβ2 plays an important role in skeletal muscle development and physiology (Grande et al., 2003). Intriguingly, the deletion of Kvβ2 in mice leads to decreased life span, increased seizures and cold-induced tremors (McCormack et al., 2002). Previously, we reported that deletion of Kvβ2 in mice led to reduce body weight, hindlimb muscle mass and size, and decreased muscle stem cells marker Pax7 protein levels, with increased Nedd4, Calcineurin, and MHC1 and MHC2a protein levels (Grande et al., 2003; Chapalamadugu et al., 2018). We also demonstrated that Kvβ2 is involved in cardiomyocyte repolarization and metabolism (Kilfoil et al., 2019), and that deletion of Kvβ2 in mice decreases isoproterenol-induced heart muscle injury and circadian clock genes (Tur et al., 2021). Therefore, we hypothesized that deletion of Kvβ2 in mice not only affects the skeletal muscle mass but also its contractile function and grip strength.

## Materials and methods

### Animals

The hemizygous Kvβ2^+/−^ null mice were a generous gift from Dr. Geoffrey Murphy (University of Michigan, MI, United States). The Kvβ2^+/−^ null mice were interbred to generate the age-matched wildtype (WT) and Kvβ2^−/−^ knockout (KO) mice as described previously (McCormack et al., 2002). All mice were fed with food and water *ad libitum*, and were maintained at Morsani College of Medicine, University of South Florida, FL, United States. The young (14–20 weeks) and old (1–2 years) WT and KO male mice were used in this study. All animal work was approved before the start of the study by the Institutional Animal Care and Use Committee (IACUC) at the University of South Florida, FL, United States.

### Grip strength

The forelimb grip strengths of 14–16 weeks and 50–58 weeks old WT and KO mice were measured with Chatillon force measurement DFE II grip strength meter (Ametek, Columbus Instruments, OH, United States) as described previously (Manickam et al., 2022). Five individual measurements were recorded per mouse and is represented as the average grip strength expressed as KGF/Kg body weight, where *n* = 6–15 mice/group.

### Myofiber force frequency relationship

The *ex vivo* contractility and force-frequency relationship (FFR) were measured as described previously (Manickam et al., 2022). Briefly, the extensor digitorum longus (EDL) muscles from both hindlimbs were collected after euthanizing the mice in compliance with the University of South Florida, IACUC guidelines. Immediately after dissecting the EDL free, it was tied with surgical suture to a force transducer in buffer containing 137 mM NaCl, 0.4 mM NaH_2_PO_4_, 5.1 mM KCl, 1.05 mM MgCl_2_, 2.0 mM CaCl_2_, and 10 mM glucose at pH 7.4 in the bath maintained at 22°C and continuously aerated with 95% O_2_/5% CO_2_. After equilibration the optimal muscle length (*l*_*0*_) for each muscle was calculated and subjected to stimulation trains (500 ms) of frequencies ranging from 1 to 125 Hz, 1 per minute to determine the force-frequency relationship. The stimulation was delivered with S48 stimulators (Grass Products, RI, United States), and all pulses were 1 ms in duration. The peak forces of each contraction at the different stimulation frequencies were used to plot the FFR. The FFR was expressed in both absolute (mN) and normalized (mN/mg muscle mass) terms, with *n* = 4–7 mice/group.

### Transmission electron microscopy

The transmission electron microscopy (TEM) images were obtained as described previously (Manickam et al., 2022). Briefly, the hindlimb EDL muscles were dissected and fixed immediately with 2.5% glutaraldehyde in 0.1 M sodium phosphate buffer for 2 h at room temperature (RT), and overnight at 4°C. Subsequent washes were carried out in phosphate buffer solution and then fixed with 1% osmium tetroxide in phosphate buffer for 2 h at 4°C. After rinsing in phosphate buffer, the muscles were then dehydrated in ethanol and acetone at RT and infiltrated in acetone: pure resin for 4 h and then embedded in the embedding wax. Polymerization of the embedding mix was carried out at 70°C overnight. 1 µm and 70 nm thin sections were cut with Reichert Ultracut microtome, stained with 2% uranyl acetate, and contrasted with lead citrate as per standard protocol. Images were obtained at x15,000, ×30,000 and ×50,000 magnifications with JOEL1400 transmission electron microscope. The myofibrillar sarcomere lengths were analyzed at ×30,000 magnified images and quantified using ImageJ software, where *n* = 3 mice/group. 7–14 images were analyzed per mouse with a total of 184 and 191 myofibrils for the young WT and KO mice, respectively. For quantification, the yellow lines were drawn from one Z-line to the next Z-line of the I bands using NIH ImageJ software to measure the length of myofibrils sarcomeres.

### Van Gieson staining

The hindlimb tibialis anterior (TA) muscles were dissected out from the young and old WT and KO mice and embedded in optimal cutting temperature (OCT) medium and frozen in isopentane chilled in liquid nitrogen and stored at −80°C. 10 µm thick transverse serial cryosections were cut at the mid-belly region using Leica (CM1860) cryostat machine and stained with Weigert’s iron hematoxylin for 10 min at RT. After washing with tap water, the sections were left in picrofuchsin solution for 20 min at RT. Washes were done with graded ethanol with picric acid, and finally with 100% ethanol until clear. The sections were then cleared with xylene for 5 min twice at RT and mounted with DPX mountant. Montage images were obtained at ×10 magnifications with Olympus IX73 inverted microscope using CellSens Standard software (Olympus Soft Imaging Solution, United States). Quantification of the fibrotic tissues area (red color) was carried out with ImageJ software (NIH, United States), where *n* = 3 mice/group. The representative images were taken at ×20 magnification.

### Quantitative real-time polymerase chain reaction

Total RNA was isolated from the hindlimb gastrocnemius (GAS) muscles of the young and old WT and KO mice using Exiqon miRCURY RNA isolation kit (Exiqon, MA, United States) as per manufacturer’s recommendation. cDNA was synthesized from the isolated total RNA using iScript cDNA synthesis kit (Bio-Rad Laboratories, CA, United States). Il6 expression was measured with iTaq Universal SYBR Green Supermix with CFX96 Real-Time C1000 Touch Thermal Cycler System (Bio-Rad Laboratories, CA, United States). The cDNA synthesis and qPCR procedures were performed as described previously (Chapalamadugu et al., 2018). The mouse GAPDH was used as an internal control reference gene, where *n* = 5–8 mice/group.

### Whole transcriptomic sequencing (RNA seq)

The hindlimb GAS muscles whole transcriptome sequencing was carried out as described previously (Manickam et al., 2022). Total RNA was extracted from the GAS muscles of young WT and KO mice, and the RNA quality and integrity were determined. RNA Seq library was then prepared. The cDNA library quality was determined before sequencing with Illumina PE150 platform. The data generated were analyzed between the WT and KO mice, where *n* = 3 mice/group for bioinformatics analysis.

### Bioinformatics

RNA-Seq data was analyzed by using two open-source software programs: TopHat and Cufflinks (Trapnell et al., 2012). Raw reads were mapped to mice GRCm38 genome by using TopHat. Cufflinks quantified genes across WT and KO groups to find differentially expressed genes (*q* value < 0.05) between these two groups. The differentially expressed genes were then entered into the Database for Annotation, Visualization, and Integrated Discovery (DAVID) (Sherman et al., 2022) to explore the KEGG gene pathways that are enriched in these genes.

### Statistical analysis

Statistical analyses were performed with GraphPad Prism 9.5.1 or SigmaPlot software. An unpaired Student’s t*-*test was utilized when comparing only two groups. A 3-way ANOVA, with 2 between-subjects factors (age and genotype) and one repeated factor (frequency) was used to test the FFR. Data are represented as mean ± SD. Statistical significance was set at *p* < 0.05 for all data analyzed either with GraphPad Prism 9.5.1 or SigmaPlot software with *post hoc* tests.

## Results

### Genetic ablation of Kvβ2 in mice results in decreased body weight

**FIGURE 1:**
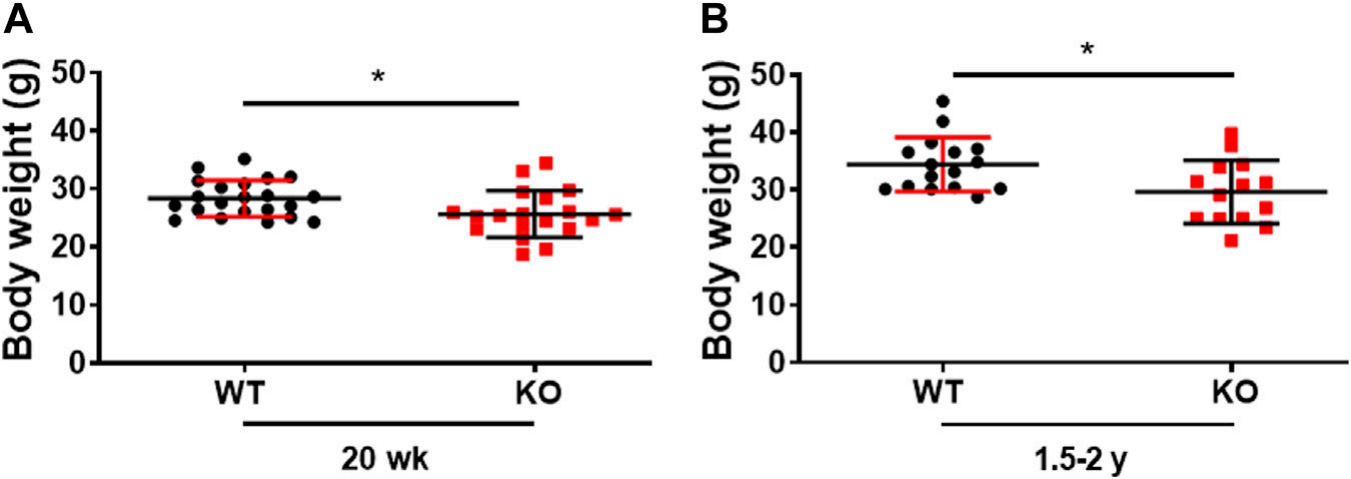
Genetic ablation of Kvβ2 in mice results in decreased body weight. **(A)** Body weight of young (20 weeks) and **(B)** old (1.5–2 years) wildtype (WT) and Kvβ2 knockout (KO) male mice in grams (g). Data are expressed as mean ± SD; *p < 0.05.

Consistent with our previous publication, the young (20 weeks) Kvβ2 KO mice displayed a significant (*p* = 0.0215) decrease in body weight compared to littermate WT mice (Chapalamadugu et al., 2018). In the present study, we demonstrate that a similar significant (*p* = 0.0161) reduction in body weight was noticed in old (1.5–2 years) Kvβ2 KO mice compared with age-matched WT mice (Figures 1A, B). Together this suggests that the deletion of Kvβ2 in mice results in decreased body weight postnatally and during aging.

### Deletion of Kvβ2 in mice results in decreased physical performance and muscle force-frequency relationship

**FIGURE 2:**
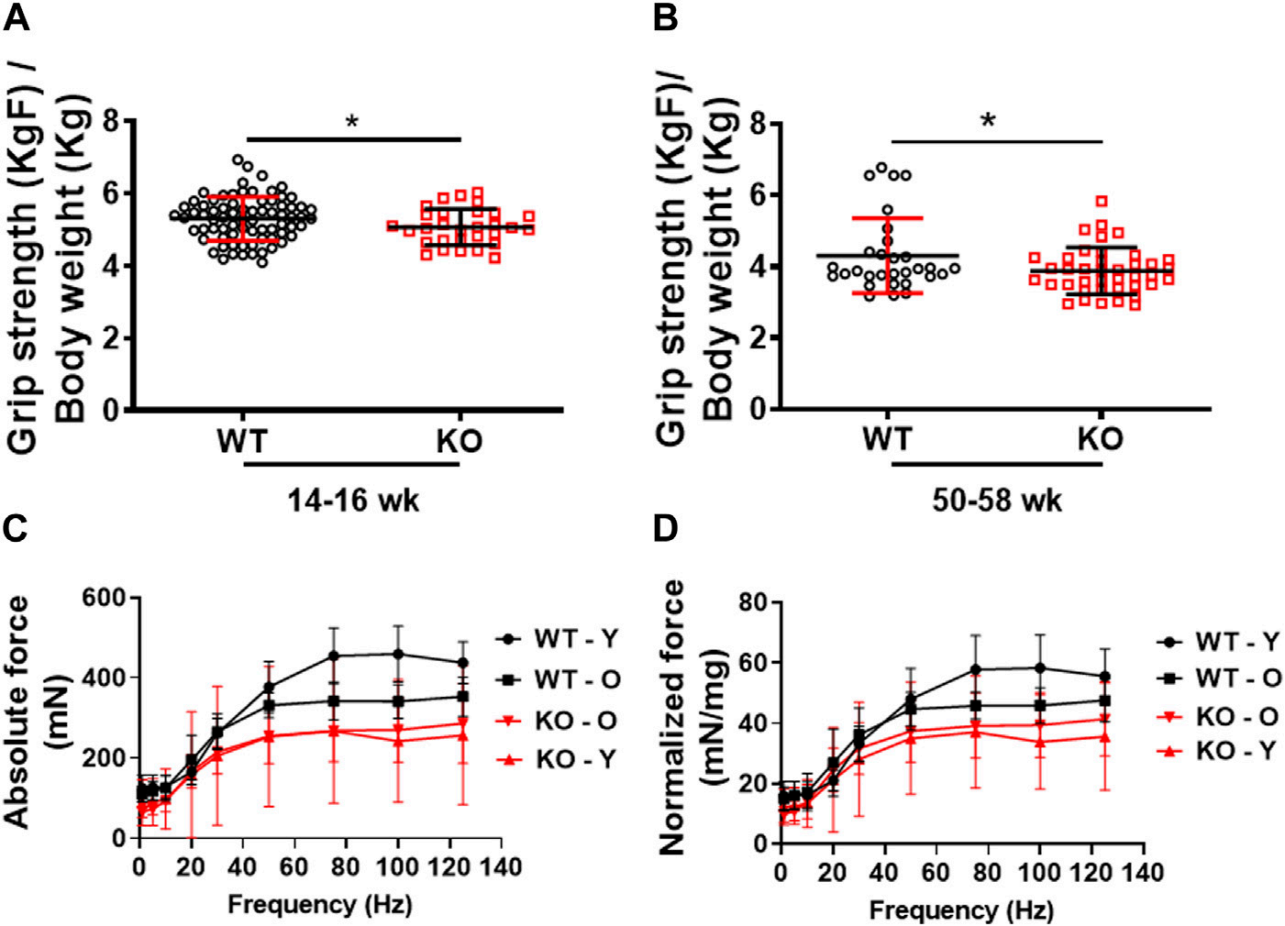
Decreased physical performance and myofiber force frequency of Kvβ2 KO mice. **(A)** Forelimb grip strength of young (14–16 weeks) and **(B)** old (50–58 weeks) wildtype (WT) and Kvβ2 knockout (KO) male mice. Data are expressed as mean ± SD; *p < 0.05. **(C)** The absolute force frequency, and **(D)** normalized force frequency of young (20 weeks) and old (2 years) WT and KO male mice hindlimb EDL muscles. The F statistics of the myofiber force frequencies with frequencies as a repeated measure displayed a significant effect on genotype (*p* < 0.001) and the age x genotype interactions (*p* = 0.006). Data are expressed as mean ± SD; where Y, Young; O, Old mice.

Previously, we demonstrated that deletion of Kvβ2 in mice results in decreased hindlimb muscles size and mass, and the muscle stem cells marker Pax7 protein levels (Chapalamadugu et al., 2018). However, it was not known if those changes resulted in any alterations in skeletal muscle contractile function and grip strength. Therefore, in the present study we assessed the forelimb grip strength and *ex vivo* skeletal muscle force-frequency relationship (FFR) of the hindlimb EDL muscles as a measure of function in the age-matched WT and KO mice. Young (14–16 weeks) Kvβ2-KO mice displayed a significant (*p* = 0.0446) decrease in forelimb grip strength compared to age-matched WT mice (Figure 2A). A similar significant (*p* = 0.0417) reduction in the forelimb grip strength was also noticed during aging in the 50–58 weeks-old Kvβ2 KO mice compared to age-matched WT mice (Figure 2B). In addition, the young (Y-20 weeks) and old (O-2yr) Kvβ2 KO mice displayed a decrease in the hindlimb EDL muscles absolute and normalized FFR compared with age-matched WT mice. There was a significant effect of genotype (*p* < 0.001), and an age x genotype (*p* = 0.006) interaction (Figures 2C, D). Together, these data suggests that deletion of Kvβ2 in mice results in impaired muscle function both *in vivo* and *ex vivo*.

### Decreased hindlimb skeletal muscle mass in Kvβ2 KO mice

**FIGURE 3:**
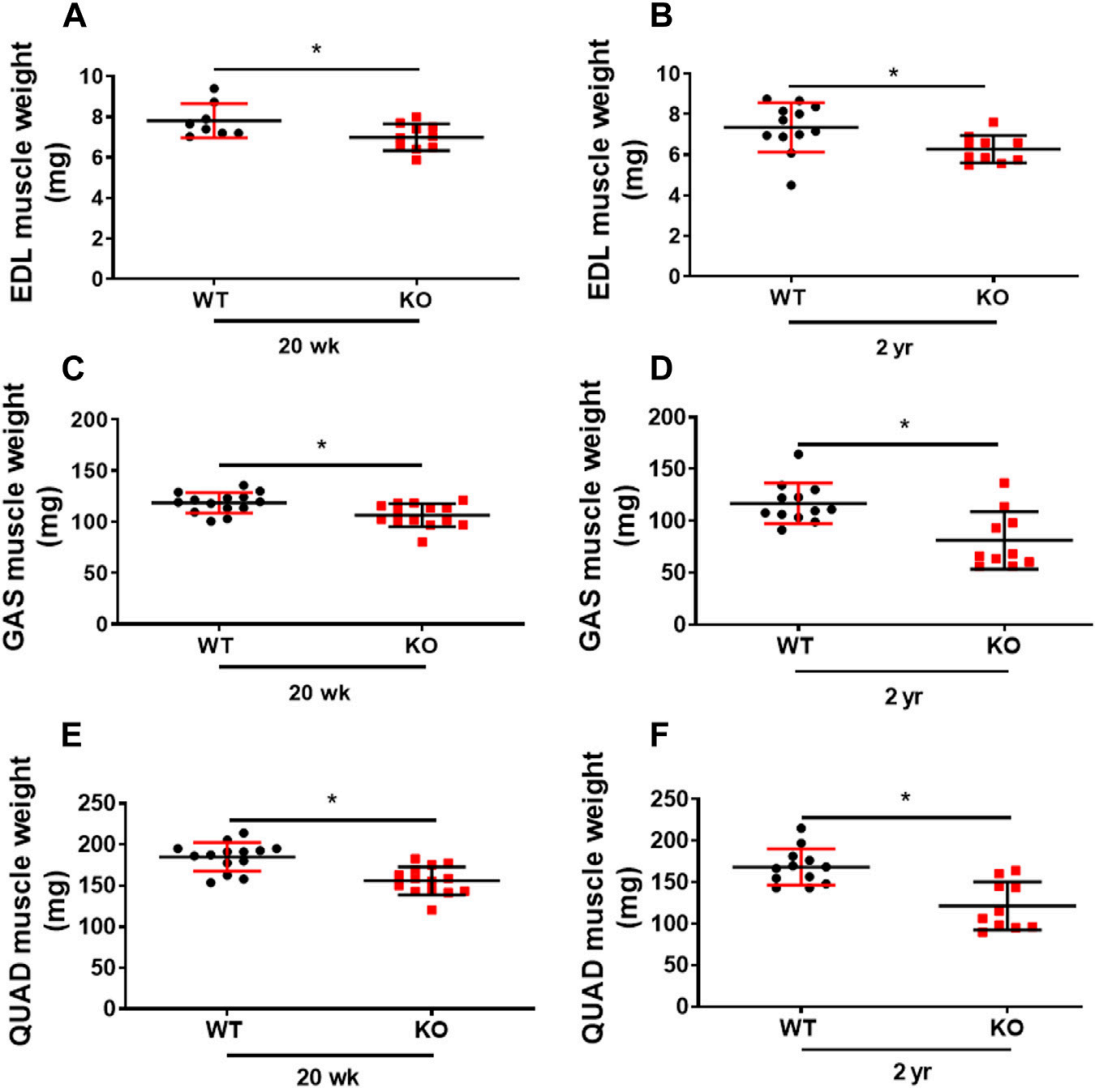
Deficiency of Kvβ2 in mice results in decreased hindlimb muscles weight. **(A)** The hindlimb EDL muscles weight of young (20 weeks) and **(B)** old (2 years) WT and KO male mice in milligrams (mg). **(C)** GAS muscles weight of young (20 weeks) and **(D)** old (2 years) WT and KO male mice in milligrams (mg). **(E)** The QUAD muscles weight of young (20 weeks) and **(F)** old (2 years) WT and KO male mice in milligrams (mg). Data are expressed as mean ± SD; *p < 0.05.

Previously, we showed that deletion of Kvβ2 in young mice results in decreased mass of hindlimb muscles: biceps femoris, gastrocnemius, and soleus (Chapalamadugu et al., 2018). Here we show a similar significant decrease not only in the young (20 weeks) Kvβ2 KO mice hindlimb muscles EDL (*p* = 0.0343), GAS (*p* = 0.0056), and Quadriceps (QUAD) (*p* = 0.0001) but also in the old Kvβ2 KO mice EDL (*p* = 0.0213), GAS (*p* = 0.0021), and QUAD (*p* = 0.0003) muscles compared to age-matched WT mice (Figures 3A–F). In addition, the direct comparison of old (2 years) WT and young (20 weeks) Kvβ2 KO mice hindlimb muscles EDL (*p* = 0.4146), GAS (*p* = 0.1065), and QUAD (*p* = 0.1169) did not show any significant difference in their weights (Supplemental Figures S1A–C). This clearly suggests that the reduction in muscle mass in Kvβ2 KO mice leads to decreased hindlimb muscles grip strengths both in the young and old mice.

### Kvβ2 regulates skeletal muscle fiber size

**FIGURE 4:**
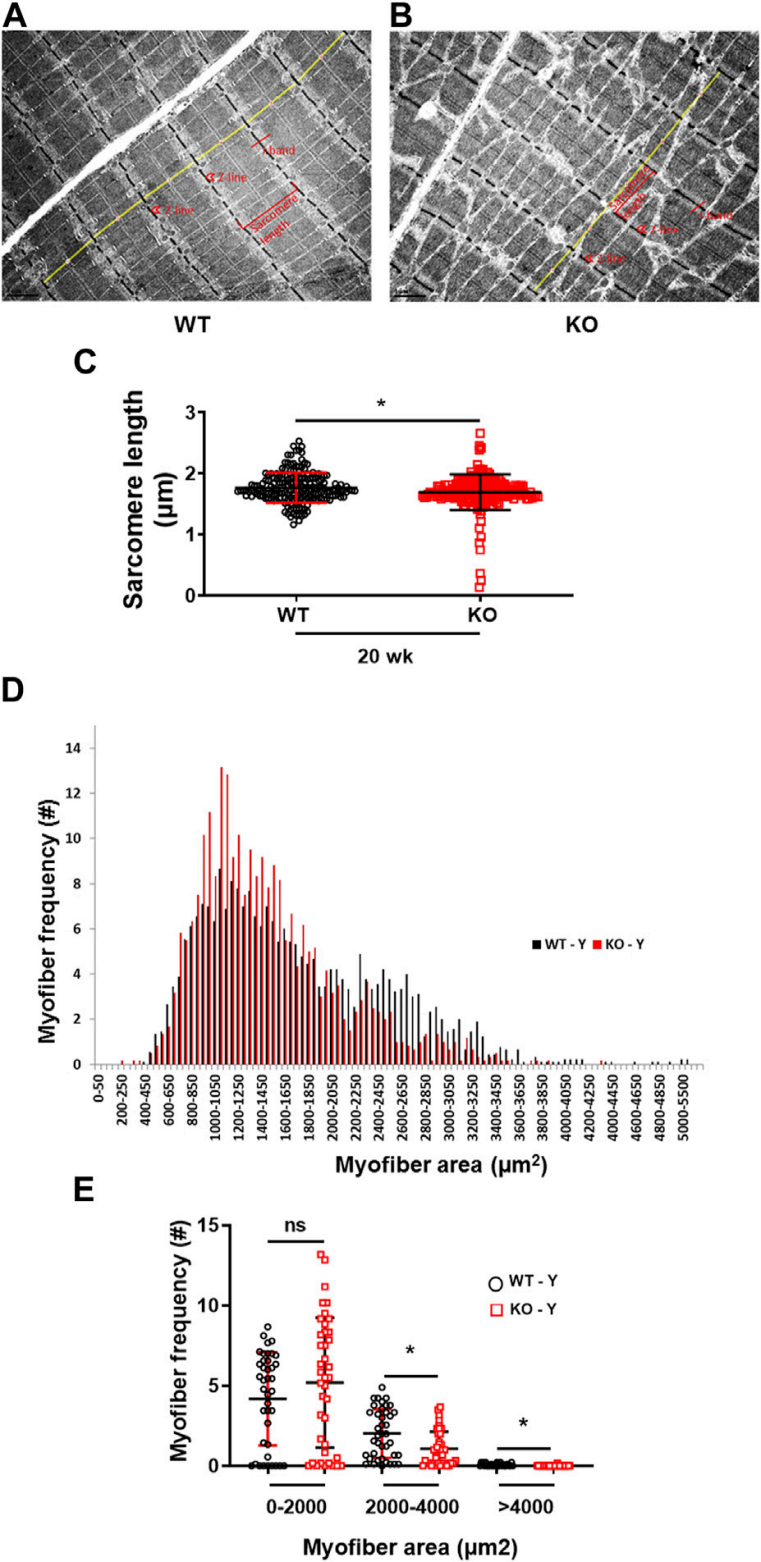
Reduced sarcomere length and myofiber size of Kvβ2 KO mice. Representative images of transmission electron microscopy of the hindlimb EDL muscles of young (20 weeks) **(A)** WT and **(B)** KO male mice. **(C)** Quantification of the EDL myofibrils sarcomere length (marked in yellow lines from one Z-line to the next Z-line [red arrow heads] of the I-bands [red lines] of the sarcomere) of WT and KO mice, where *n* = 184 and 191, respectively. **(D,E)** Quantification of 250 random myofibers cross-sectional areas of hematoxylin and eosin-stained TA muscles, where *n* = 3 mice/group x 3 sections/mouse. Data are expressed as mean ± SD; *p < 0.05; ns, non-significant.

The transmission electron microscopy image analysis of the longitudinal sections of the hindlimb EDL muscles displayed a significant (*p* = 0.0086) reduction in myofibrils sarcomere length in the young (20 weeks) Kvβ2 KO mice compared to age-matched WT mice (Figures 4A–C). In addition, a significant reduction in the myofiber cross-sectional area of the medium (2,000–4,000 µm^2^) (*p* = 0.0016) and largest (>4,000 µm^2^) (*p* = 0.0009) sized myofiber areas was also observed in the young (20 weeks) Kvβ2 KO mice hindlimb TA muscles cryosections stained with hematoxylin and eosin (H&E) compared to age-matched WT mice (Figures 4D, E), These results further confirm that deletion of Kvβ2 in mice results in decreased hindlimb muscle mass.

### The young Kvβ2 KO mice phenocopy inflammaging phenotype

**FIGURE 5:**
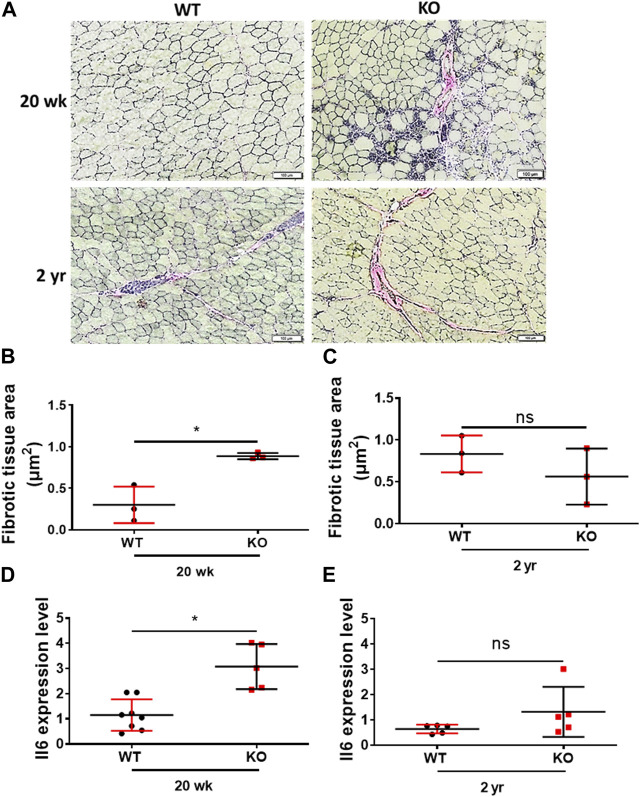
Deletion of Kvβ2 results in increased skeletal muscle tissue fibrosis in young mice. **(A)** Representative ×20 magnification images of the hindlimb TA muscles cross-sections at mid-belly region of young (20 weeks) and old (2 years) WT and KO male mice stained with Van Gieson stain. **(B)** Quantification of fibrotic tissues area (red color) of young (20 weeks) and **(C)** old (2 years) WT and KO male mice stained with Van Gieson stain, where *n* = 3 mice/group whole sections. **(D)** RT-qPCR analysis of Il6 expression levels in young (20 weeks) and **(E)** old (2 years) WT and KO male mice, where *n* = 5−8 mice/group. Data are expressed as mean ± SD; *p < 0.05, ns, non-significant.

We then assessed the inflammaging phenotype of the skeletal muscles of the young (20 weeks) and old (2 years) mice through Van Gieson staining (fibrosis) of the hindlimb TA muscles cryosections and expression of the proinflammatory marker Il6 through RT-qPCR. Deletion of Kvβ2 displayed a significant (*p* = 0.0103) increase in fibrotic tissue area in the young (20 weeks) mice compared to age-matched WT mice, whereas it was found to be non-significant (*p* = 0.3081) in the old mice between the genotypes (Figures 5A–C). This was accompanied by a significant (*p* = 0.0008) increase in the proinflammatory marker Il6 expression in the young (20 weeks) Kvβ2 KO mice compared to age-matched WT mice (Figure 5D). However, the expression levels Il6 was found to be non-significant (*p* = 0.1699) in the 2 years old WT and KO mice (Figure 5E).

### Deletion of Kvβ2 in mice results in enhanced skeletal muscle atrophy, inflammation, altered metabolism, and circadian clock genes expression

**FIGURE 6:**
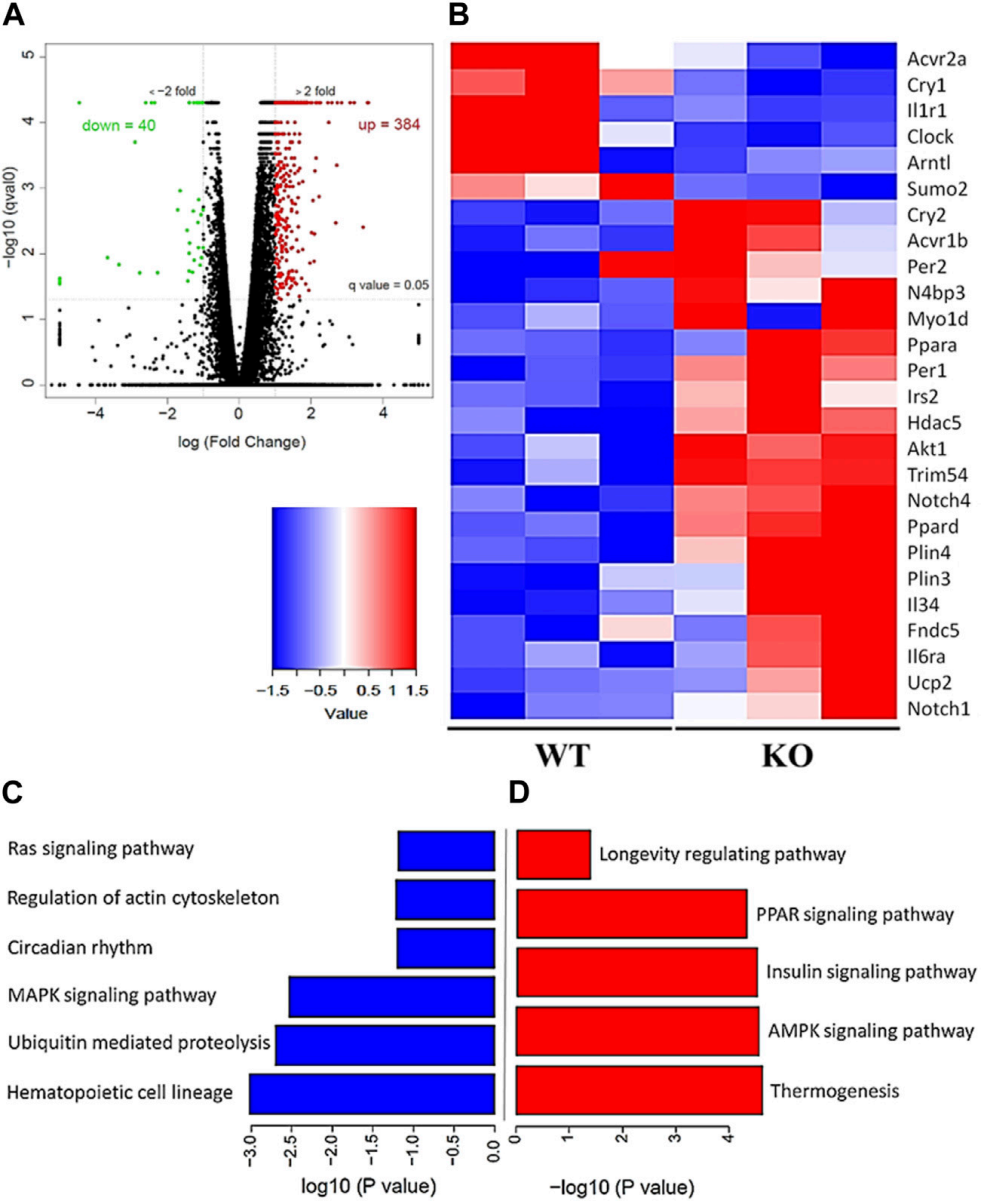
RNA Seq data analysis of the hindlimb GAS muscles of young (20 weeks) WT and KO mice. **(A)** Volcano plot showing differentially expressed genes in the KO_vs._WT mice. **(B)** Heat map of the key genes that are differentially expressed in the KO_vs._WT mice. **(C)** Signaling pathways that are downregulated and **(D)** upregulated in the KO_vs._WT mice. Data are expressed as significantly different where *q* < 0.05, and *n* = 3 mice/group.

To evaluate the differential gene expression of the young Kvβ2 KO and WT mice, RNA Seq analysis was performed in the hindlimb GAS muscles. The volcano plot shows the number of differentially expressed key genes (Figure 6A) that were significantly (*p* < 0.05) upregulated (384 genes) and downregulated (40 genes) in the young Kvβ2 KO mice compared to age-matched WT mice. Subsequent heat map analysis of the differentially expressed key genes in the young Kvβ2 KO and WT mice displayed a significant (*p* < 0.05) increase in the expression of genes involved in skeletal muscle development, cell proliferation and determination (*Myo1d, Notch1*, and *Notch4*), muscle atrophy (*Trim54, Hdac5, Sumo2, Acvr1b,* and *N4bp3*), energy metabolism and muscle plasticity (*Irs2, Akt1, Ucp2, Fndc5, Plin3, Plin4, Ppara,* and *Ppard*), and inflammation (*Il6ra* and *Il34*), and a decrease in circadian core clock (*Clock* and *Arntl*) genes along with an increase in the negative feedback loop clock gene repressors such as *Per1, Per2* and *Cry2* in young Kvβ2 KO mice compared to age-matched WT mice. However, there was also a decrease in *Acvr2a, Cry1* and *Il1r1* genes expression in the young Kvβ2 KO mice compared to age-matched WT mice (Figure 6B) suggesting the differential regulation of various signaling pathways involved in muscle atrophy, peripheral circadian rhythm and inflammation between the genotypes (Figures 6C, D).

## Discussion

The voltage-gated potassium channel β2 subunit (Kvβ2) has been implicated in many physiological functions, however, its role in skeletal muscle function remains unknown. Therefore, in the present study we sought to test the effect of Kvβ2 deletion on mouse skeletal muscle function. We report three major changes in the mouse skeletal muscle based on the deletion of Kvβ2 in mice: 1) deletion of Kvβ2 leads to decreased muscle function, 2) genetic deletion of Kvβ2 leads decreased myofibrils sarcomere length and myofiber cross-sectional area and 3) RNA seq analysis provides insights into the differential gene expression patterns that are consistent with muscle atrophy, inflammation, altered energy metabolism and circadian rhythm.

Based on the study, we demonstrate that there was reduced forelimb grip strength in the young Kvβ2 KO mice, which was also apparent during aging in these mice. Previously, we reported that the deletion of Kvβ2 in mice leads to reduced hindlimb muscle and body weights with decreased Pax7 protein levels during postnatal growth (Chapalamadugu et al., 2018). Here, we confirm a robust reduction in the hindlimb muscles and body weights in young Kvβ2 KO as well as in old Kvβ2 KO mice. Consistent with our previous finding, we also found a similar reduction in the medium to largest diameter sized myofibers area in the hindlimb TA muscles cross-sections of the young Kvβ2 KO mice analyzed (Chapalamadugu et al., 2018). Similarly, the transmission electron microscopy image analysis of the hindlimb EDL muscles longitudinal sections also revealed a decrease in the myofibrils sarcomere length in the young Kvβ2 KO mice suggesting plausible myofiber atrophy. Together, this suggests that the observed decline in the muscle function might be due to its direct association with the decreased muscle weights. To address this question, we utilized the *ex vivo* hindlimb EDL myofibers force-frequency relationship and found that the deletion of Kvβ2 in mice leads to decreased absolute and normalized EDL myofibers FFR both in the young and old Kvβ2 KO mice, suggesting that muscle function is compromised by loss of muscle mass as well as the decreased length of the sarcomere.

Intriguingly, the young Kvβ2 KO mice displayed an increase in fibrotic tissue area in hindlimb TA muscles cross-sections stained with Van Gieson’s staining but was found to be non-significant between the genotypes in the old mice as the aged WT mice also displayed an increase in the fibrotic tissue area consistent with the aging muscle phenotype. To test this, we performed an in-depth RNA-Seq analysis of the differentially expressed genes in the hindlimb GAS muscles of the young Kvβ2 KO_vs._WT mice. The volcano plot analysis identified 384 and 40 genes to be up- and downregulated in the Kvβ2 KO mice, respectively. Subsequent, heat map analysis revealed an increase in the expression of number of key genes involved in skeletal muscle development, proliferation and cell fate determination (Notch signaling), atrophy (Neurogenic), inflammation (Interleukins), energy metabolism and muscle plasticity (Ppars and Insulin signaling), along with a decrease in the circadian peripheral core clock genes (*Clock* and *Bmal1/Arntl*) and an increase in the negative feedback loop core clock gene repressors (*Per* and *Cry*) reflective of impaired muscle phenotype (Luo et al., 2005; Perera et al., 2012; Manickam and Wahli, 2017). Noticeably, we showed previously the downregulation of circadian core clock *Bmal1* gene expression in the cardiac tissue samples of the young Kvβ2 KO mice (Tur et al., 2021). In addition, we also validated the pro-inflammatory *Il6* myokine expression through real-time quantitative PCR analysis of the hindlimb GAS muscles of the young and old Kvβ2 KO mice and found that *Il6* expression was higher in both the young and old Kvβ2 KO mice.

Previously, we showed that deletion of Kvβ2 in mice enhanced NEDD4 levels and decreased PAX7 protein levels in skeletal muscle during postnatal myogenesis (Chapalamadugu et al., 2018) suggesting that Nedd4 might negatively regulate *Pax7* expression in the young Kvβ2 KO mice. In this study we show an increase in NEDD4 ubiquitin ligase binding protein 3 (*N4bp3*) expression in the young Kvβ2 KO mice. We also found that deletion of Kvβ2 in mice leads to an increase in *Myo1d* (Myosin1d), along with an increase in *Notch* 1 and 4 expression levels in the Kvβ2 KO mice, suggesting that there might be an increase in the early progenitor population in these mice (Luo et al., 2005). In addition, we also noticed an increase in the *Acvr1b*, *Hdac5*, and *Trim54/MuRF3* expression levels in the Kvβ2 KO mice suggesting possible activin receptor signaling, and neurogenic muscle atrophy of skeletal muscles in these mice leading to reduced muscle mass, grip strength, the contractile unit sarcomere length, myofiber size, and increased fibrosis (Moresi et al., 2010; Lee et al., 2020). Furthermore, the circadian core clock genes *Clock* and *Arntl/Bmal1* expression was found to be downregulated in the Kvβ2 KO mice with a simultaneous increase in the negative feedback loop core clock gene repressors *Per1*, *Per2*, and *Cry2* expression levels, which clearly indicates the involvement of peripheral circadian clock genes in the Kvβ2 KO mice (Sadria and Layton, 2021). There was also an increase in the pro-inflammatory markers such as *Il34*, *Il6ra*, and a decrease in the *Il1r1* agonist/antagonist receptor suggesting that there is increased inflammation in the skeletal muscles of the young Kvβ2 KO mice. Additionally, we also noticed an increase in the expression of genes involved in energy metabolism and muscle plasticity such as *Ppars* (*Ppar-α* and *Pparδ*) and insulin (*Irs2/Akt1*) signaling, lipid droplets associated proteins (*Plin3, 4*), browning of adipose tissues (*Fndc5, Ucp2*), and a decrease in the small ubiquitin-like modifier 2 (*Sumo2*) in the young Kvβ2 KO mice. Interestingly, there was also an increase in the longevity associated pathway, i.e., the NAD^+^-dependent protein deacetylase *Sirt6* expression in the Kvβ2 KO mice, which is implicated in cellular stress resistance, genomic stability, aging and energy homeostasis (Roichman et al., 2021). Collectively, these data suggest that the genetic ablation of Kvβ2 in mice results in the use of alternate energy metabolism to overcome the overt skeletal muscle phenotype observed in young adults.

## Conclusion

The present study demonstrates that deletion of Kvβ2 decreased muscle mass and strength. The deletion of Kvβ2 in mice also resulted in increased fibrotic tissues area in the young Kvβ2 KO mice along with an increase in the proinflammatory markers. RNA Seq analysis showed increased expression of genes involved in muscle atrophy, energy metabolism, inflammation, and circadian rhythm. Overall, this is the first study linking kcnab2 gene to age related muscle function along with molecular insights into signaling pathways.

## Data Availability

The datasets presented in this study can be found in online repositories. The names of the repository/repositories and accession number(s) can be found in the article/Supplementary Material. GEO accession number GSE233996.

## Supplementary material

Supplemental Figure S1 Comparison of hindlimb muscle weights in 2-year-old WT and 20-week young Kvβ2 KO mice. **(A)** EDL muscles weight. **(B)** GAS muscles weight. **(C)** QUAD muscles weight. Data are expressed as mean ± SD; ns, non-significant.
