# Supplementary figures and images for: Genetic deletion of Kvβ2 (AKR6) causes loss of muscle function and increased inflammation in mice
Source: Front Aging. 2023 Jun 12;4:1175510. doi: 10.3389/fragi.2023.1175510 (PMC10292803; doi:10.3389/fragi.2023.1175510)

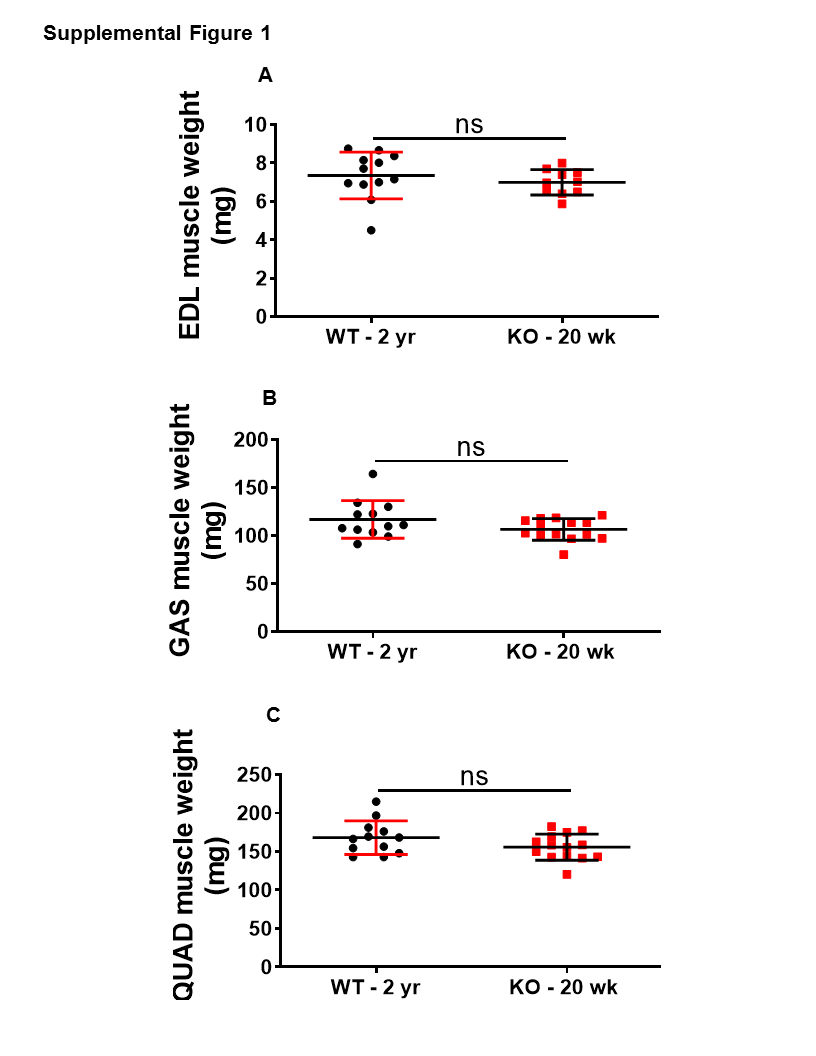

Supplement: Supplementary file 1 [file Image1.tif]
